# Complete plastid genome of *Lespedeza tricolor* (Fabaceae), an endemic shrub in Korea

**DOI:** 10.1080/23802359.2022.2130714

**Published:** 2022-10-16

**Authors:** Tae-Hee Kim, Sang-Chul Kim, Young-Ho Ha, Kyung Choi, Hyuk-Jin Kim

**Affiliations:** Division of Forest Biodiversity, Korea National Arboretum, Pocheon-si, Republic of Korea

**Keywords:** Plastid genome, *Lespedeza tricolor*, Korean endemic species, phylogenetic relationship

## Abstract

*Lespedeza tricolor* is a species found in the southern province of Korea, thought to be endemic to Korea. The complete plastid genome of this legume was sequenced in this study. DNA from *L. tricolor* was extracted, sequenced, and assembled into the complete plastid genome. We used 18 species of the family Fabaceae and 77 protein-coding genes to perform phylogenetic analysis. The plastid genome is 149,038 bp in length, with large (82,495 bp) and small (18,889 bp) single-copy regions, separated by a pair of inverted repeat regions (23,827 bp). It contains 83 protein-coding genes, eight rRNAs, 37 tRNAs, and two pseudogenes (*rpl22* and *infA*). Our phylogenetic analysis suggests that the genus *Lespedeza* is monophyletic and *L. tricolor* is closely related to *L. maritima* and *L. buergeri*. In this study, we identified the phylogenetic position of *L. tricolor* and provided the data that can be used in various ways in future studies.

The genus *Lespedeza* Michx. (tribe Desmodieae; subfamily Papilionoideae; family Fabaceae), which comprises approximately 60 species, has the distribution of Asia and North America (Han et al. 2010). Due to their structure and characteristics, these species are used as forage and medicine, or for ornamental purposes (Cheng et al. 2004; Guan et al. 2013; Sharma and Rhyu 2015; Somaratne et al. 2019). In South Korea, 25 species have been recognized (Korea National Arboretum 2022). Among them, *Lespedeza tricolor* (Nakai) D. P. Jin, J. W. Park & B. H. Choi 2019 is an endemic species to Korea and inhabits the southern province (Jin et al. 2019). Many suggestions have been made on the taxonomic position of *L. tricolor*, such as, as a variety of *L. maximowiczii* [= var. *tricolor* Nakai], subspecies of *L. buergeri* [= subsp. *tricolor* (Nakai) Hatusima], or a synonym of *L. maximowiczii* (Hatusima 1967; Akiyama 1988; Jin et al. 2019). Jin et al. (2019) using microsatellite data analysis showed that although *L. tricolor* formed mixed lineages with *L. maximowiczii* and *L. buergeri*, it was a distinct species. To clarify the phylogenetic relationships of *L. tricolor* and related species, this study aims to determine the complete plastid genome sequence of *L. tricolor* and conduct phylogenetic analysis using 77 plastid protein-coding gene datasets.

Fresh leaves of *L. tricolor* were collected from Wando-gun, Jeollanam-do province, South Korea (34°20′25.0″N 126°41′37.6″E) and dried directly with silica gel at room temperature until DNA extraction. The voucher specimen was deposited in the herbarium of the Korea National Arboretum (KH; http://www.nature.go.kr/kbi/plant/smpl/KBI_2001_030100.do, Hee Young Gil, E-mail: warmishe@korea.kr, voucher number: ESK21-503). Total genomic DNA was extracted using DNeasy Plant Mini Kit (Qiagen Inc., Valencia, CA). Next-generation sequencing was conducted using MiSeq sequencing system (Illumina, Seoul, South Korea) and a total of 10,049,414 reads were obtained. The GetOrganelle software was used to select the plastid-like reads, which were assembled into the complete plastid genome by Geneious Prime program and the GetOrganelle toolkit (Kearse et al. 2012; Jin et al. 2020). Gene content and order were annotated using the Geseq tool and Geneious Prime (Kearse et al. 2012; Tillich et al. 2017).

To identify the phylogenetic relationships of subfamily Papilionoideae, a total of 18 species were used, of which three species (*Acacia ligulata*, GenBank accession = NC_026134; *Erythrophleum ivorense*, GenBank accession = MZ274091; *Ceratonia siliqua*, GenBank accession = NC_026678) were designated as outgroups. For phylogenetic analysis, 77 protein-coding genes were aligned using the MAFFT program and maximum-likelihood (ML) analysis in the software PhyloSuite (Katoh and Standley 2013; Zhang et al. 2020). The best substitution model was TVM + F+R2 according to ModelFinder in PhyloSuite (Zhang et al. 2020). 1000 bootstrap (BS) replications were performed based on a dataset of protein-coding genes.

The complete plastid genome of *L. tricolor* (GenBank accession = ON227501) is 149,038 base pairs (bp) long, with a typical quadripartite structure which has components such as a large single-copy (LSC) of 82,495 bp, a small single-copy (SSC) of 18,889 bp, and two inverted repeats (IRs) of 23,827 bp. The total GC content was 35.0%, and the GC contents of the LSC, SSC, and IR regions were 32.4%, 28.2%, and 42.2%, respectively. The plastid genome includes 128 genes (83 protein-coding genes, eight rRNAs, and 37 tRNAs) including 17 genes (six protein-coding genes, four rRNAs, and seven tRNAs) duplicated in the IR regions. Two genes (*rpl22* and *infA*) were missed in *L. tricolor* (Millen et al. 2001; Jansen et al. 2008; Magee et al. 2010). In addition, the tribe Desmodieae is known to have no introns within *rps12* and *rpl2* (Jansen et al. 2008). This study also confirmed the absence of introns for these genes in *L. tricolor*.

The ML trees showed that Papilionoideae were monophyletic with high BS support values (100) and *L. tricolor* was a sister group of *L. maritima* and *L. buergeri* (Figure 1). The complete plastid genome of *L. tricolor* will be useful to study the phylogeny, develop identification markers, and understand the evolutionary history for *Lespedeza* species.

**Figure 1.**
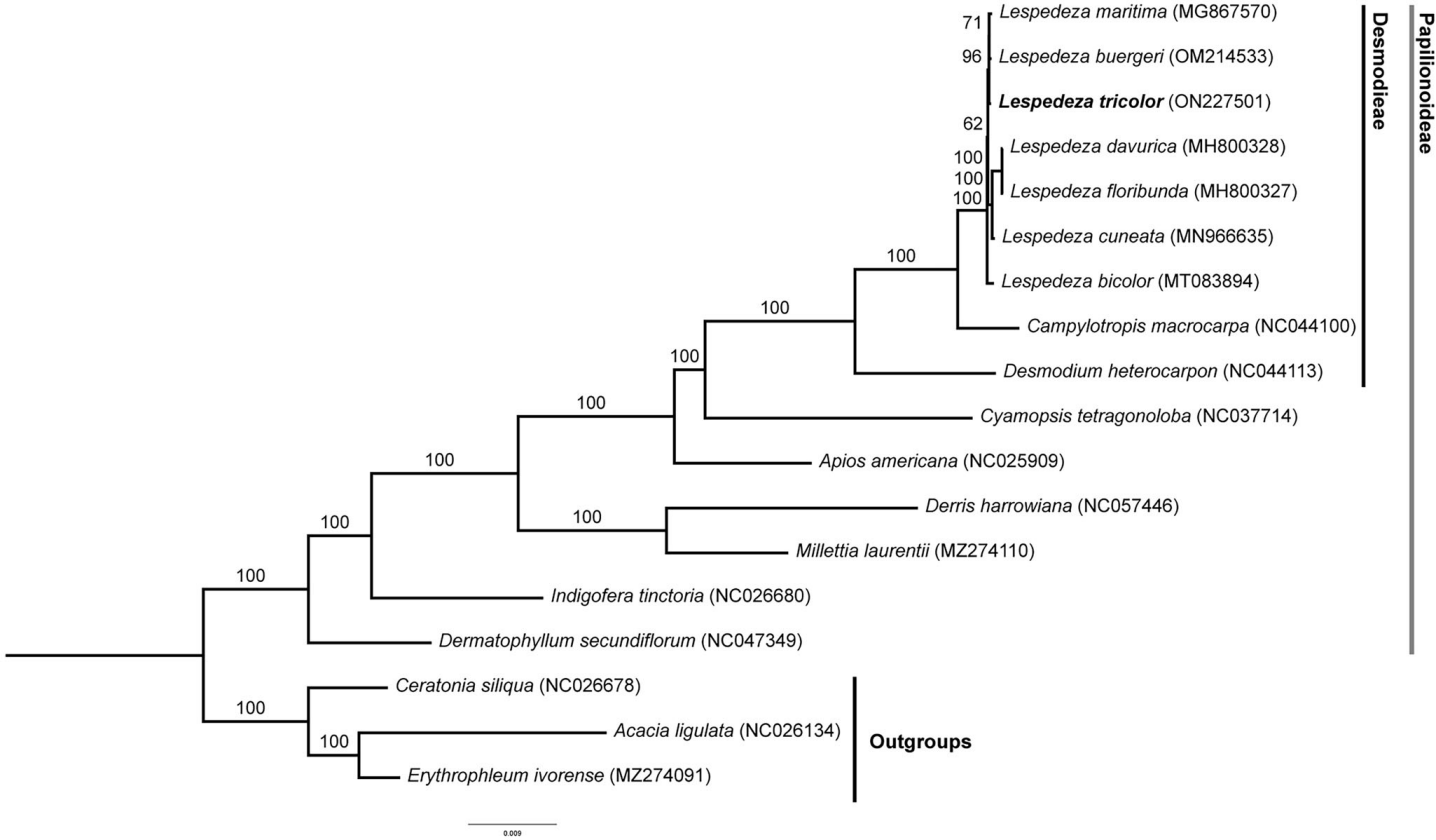
Maximum-likelihood tree of 18 legumes inferred from combining 77 protein-coding genes. The numbers above each node are the bootstrap support values from 1000 replications.

## Ethical approval

The material involved in the paper does not involve ethical conflicts. Therefore, it requires no specific permissions or licenses.

## Author contributions

Hyuk-Jin and Kyung conceived the original structure of the review. Sang-Chul and Young-Ho have collected the data and conducted the experiments. Tae-Hee analyzed the data and drafted the manuscript. All authors have read and agreed to the submitted version of the manuscript.

## Data Availability

The data that support the findings of this study are openly available in NCBI at https://www.ncbi.nlm.nih.gov/ (reference number ON227501). The associated BioProject, SRA, and Bio-Sample numbers are PRJNA826218, SRR18762682, and SAMN27553414, respectively.
